# Bifurcation structure of adaptation versus depolarization block

**DOI:** 10.1186/1471-2202-13-S1-P169

**Published:** 2012-07-16

**Authors:** Kun Qian, Marco Antonio Huertas Chacon, Carmen Canavier

**Affiliations:** 1 Neuroscience Center of Excellence, Louisiana State University Health Science Center, New Orleans, LA, USA

## 

**Figure 1:**
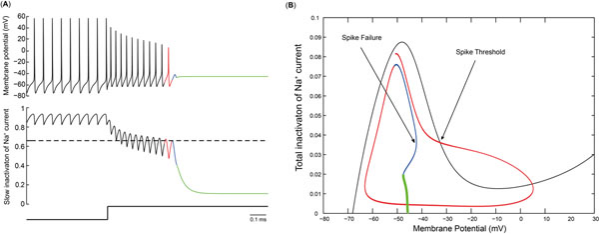
Spike Failure via Adaptation. **(A)** Time course of the membrane potential (top) and slow Na^+^ channel inactivation, middle), during spike failure after a depolarizing current step (bottom). **(B)** Phase plane for fast/slow analysis. The N shaped voltage nullcline (black curve) is calculated at a constant value of slow channel inactivation corresponding to spike failure (dashed line in panel (A)). The middle branch is the effective spike threshold. The red trajectory corresponding to the red waveform in the left two traces exceeds this threshold but not the blue one.

Midbrain dopamine neurons fire in a pacemaker-like fashion in vitro where they are deprived of most afferent input. In vivo, these neurons can emit a burst of action potentials with fast (~50 Hz) frequencies [1]. However, simple somatic depolarization of these neurons does not elicit such rapid firing because the neurons cease firing at levels of depolarizing somatic current injection stronger than those that elicit repetitive firing at about 10 Hz [2]. Here we argue that in the case of simple somatic current injection, firing ceases due to adaptation rather than depolarization block. We hypothesize that the primary adaptation mechanism is the slow inactivation of the fast sodium current [3], which causes the spike threshold to become more depolarized with each spike until spiking fails. We contrast the bifurcation structure of a simple 3 variable model that ceases to fire via this mechanism with a two variable model that fails by going into depolarization block. In both cases, spontaneous firing is characterized by an N shaped voltage nullcline with two stable branches. For the depolarization block, firing ceases when the fixed point moves from the unstable to the stable branch at the knee of the nullcline (Fig.1). The action potentials decrease in amplitude both at the peak and the trough, and the resulting stable potential is much more depolarized than the spike threshold for the adaptation case. In contrast, for the adaptation case, only the peaks of the action potential decrease slightly, and the final stable potential is at the spike threshold. As slow inactivation proceeds, the voltage nullcline loses the unstable middle branch, and the two stable branches merge. Spiking fails when the trajectory no longer crosses the unstable middle branch (the spike threshold) because the increasing ISI length allows for more fast inactivation. The two mechanisms can be differentiated experimentally: an additional depolarizing square pulse of current applied just after the last spike should evoke an additional action potential for adaptation, but not depolarization block.
